# Influence of Different Hardness Custom Foot Insoles in the Electromyography Activity Patterns of the Thigh and Hip Muscles during Motorcycling Sport: A Crossover Study

**DOI:** 10.3390/s20061551

**Published:** 2020-03-11

**Authors:** Israel Casado-Hernández, Ricardo Becerro-de-Bengoa-Vallejo, Marta Elena Losa-Iglesias, Eva María Martínez-Jiménez, Daniel López-López, Victoria Mazoteras-Pardo, Carlos Romero-Morales, César Calvo-Lobo

**Affiliations:** 1 Faculty of Health Sciences, Universidad Rey Juan Carlos, 28922 Alcorcón, Spain; israelcasado@yahoo.es (I.C.-H.); marta.losa@urjc.es (M.E.L.-I.); 2 Facultad de Enfermería, Fisioterapia y Podología, Universidad Complutense de Madrid, 28040 Madrid, Spain; ribebeva@ucm.es (R.B.-d.-B.-V.); vmazoter@ucm.es (V.M.-P.);; 3 Facultad de Fisioterapia y Enfermería, Departamento de Enfermería, Universidad de Castilla la Mancha, 45071 Toledo, Spain; Eva.Martinez@uclm.es; 4 Research, Health and Podiatry Group, Department of Health Sciences, Faculty of Nursing and Podiatry, Universidade da Coruña, 15403 Ferrol, Spain; daniel.lopez.lopez@udc.es; 5 Faculty of Sport Sciences, Universidad Europea de Madrid, Villaviciosa de Odón, 28670 Madrid, Spain

**Keywords:** insoles, surface electromyography, muscular disease, motorcycle, support

## Abstract

Nowadays, the use of insoles in sport practice have been recognized to decrease the foot and lower limb injury patterns. The aim of this study was to analyse the effect of four types of hardness insoles (HI) in the activity patterns of the hip and thigh muscles (HTM) in motoriders during motorcycling sport. The study was a crossover trial. Subjects were elite motoriders. The mean age was 33 ± 5.14 years. Electromyography (EMG) of hip and thigh muscles (HTM) data was registered via surface while subjects were riding on an elite motorcycle simulator. Subjects had to complete different tests with randomly hardest insoles (HI): 1: only polypropylene (58° D Shore); 2: Polypropylene (58° D Shore) with selective aluminium in hallux and metatarsal heads (60 HB Brinell hardness); 3: Ethylene vinyl acetate (EVA) (52° A Shore); and finally, 4: Ordinary EVA (25° A Shore) as the control. EMG patterns of the HTM, riding on an elite motorcycle simulator, showed the lowest peak amplitude with the insoles with polypropylene and selective aluminium. Using the hardest insoles in our study (selective aluminium) the EMG amplitude peaks decreased in all HTM.

## 1. Introduction

Motorider performance in speed circuit race arises from various elements: motorider physical condition, motorcycle elements composition: tires, suspensions, brakes, chassis, engine and, finally, environmental factors. Competing motorcycles and speed circuits are not identical, and the hip and lower limb muscular fatigue influence in riders remains unmeasurable. Published research focusing on biomechanical and muscular fatigue in riders is relatively sparse [1,2]. Motoriders undergo a high physical load while they quickly accelerate and abruptly decelerate. They attempt to gain control of the centre of mass distribution of the motorcycle in various riding requirements, for example, when a motorider is negotiating straights and turns [3,4].

Muscular tiredness is a very complex phenomenon. It can be depicted as a time-dependent practice induced depletion in the maximum contraction force of each muscle [5]. Loss of strength depends on the duration and intensity of practise and on the kind of muscular shrinkage [6]. Research has focused on upper limb riders injuries [7,8,9]. Hip and thigh muscles play a suitable role across the forces made by the feet on the footpeg in motorcycling performance [10]. Currently, we have found in the scientific literature recent studies that describe the importance and influence of the motorider physical condition on the performance riding on a motorcycle. A poor physical condition produce an increase in muscle fatigue and, therefore, a performance decrease. Likewise, sudden muskuloesqueletal movements riding on the motorcycle with a deficient physical condition will produce injuries [11]. Highlight Achilles tendinophaty, patellofemoral syndrome, shallow soft tissue, knee pain, due to sudden changes on the motorcycle and plantar injury like paraesthesia due to an increase of plantar pressure on the footpeg [11,12,13,14].

Nowadays, the impact of the prevalence and complaints rate of HTM alterations showed an estimated range around of 10% and 40% that commonly appear in the practice of the various types of bicycles that include: 1: bike motocross, 2: motorcycling sport, 3: road bike, 4: stationary cycles and 5: stunt bicycles [13].

These injuries present an assortment of multifactorial reasons associated with: Abnormality of HTM, musculoesqueletal disturbance, features of riding surroundings, male genus morphological characteristics, the kind of boot and foot pedal strength, which continue without be clear due to the complex structure and factors that involve this problem [1,15].

Nonetheless, any prior investigation has evaluated this issue related to EMG patterns in riders during the motorcycling sport and the effects of different HI in the activity of HTM. These tools are generally utilized to assess muscle patterns activity in the cycling. Preceding reports recorded meaningful oscillations in the EMG patterns of HTM during the praxis of cycling sports [14,16,17].

Based on these depositions, the principal purpose of our investigation was to evaluate the effect of four types of hardness CFI in the activity patterns of the hip and thigh muscles (HTM) in healthy people during motorcycling sport. The hypothesis in our inquiry is that participants wearing different HI performing motorcycling may display variations in the EMG of the HTM. The principal main goal is to determinate which material of the HI decrease EMG patterns.

## 2. Materials and Methods

### 2.1. Design and Sampling

Nine male healthy motorcycle riders participated in outpatient foot specialist clinic, in the town of Madrid (Spain) from November to December 2018. A crossover study (prospectively registered in ClinicalTrials.gov as NCT03734133 on November 28, 2018) was carried out and a non-random consecutive sampling method was applied to recruit all participants. The inclusion criteria comprised: (1) at least eighteen years old, (2) healthy motorcycle riders without medical problems in the clinical record neither family history with relevant illness and (3) riders who understood and signed the informed consent document for participation in our research. Additionally, the exclusion criteria included: (1) medical trauma and a history of leg problems, (2) musculoskeletal disturbances, (3) vascular diseases, (4) refusal to sign and deliver consent informed document and (5) an inability to understand the protocol to perform our research. Data were collected as previously describe in [18]. 

Other hand, our study was accomplished succeeding the guidelines and list for Template for Intervention Description and Replication (TIDieR) [19].

### 2.2. Method

At first time a preliminary evaluation was focused on a general physical condition, socio-demographic data composed by (1) age, (2) sex, (3) height, (4) weight, (5) Body Mass Index (BMI) and (6) foot size, chronic diseases with dysfunction like musculoskeletal disorders and, finally, enjoyment activities. A specialized motorcycle sport podiatrist physically evaluated each subject and took the measurement of anthropometric variables, like height, weight, and BMI.

A professional motorcycle simulator was used in our study. The simulator has the shape of a sport motorcycle and a television screen was at the front of the simulator playing a real motor racing. A video of a speed circuit is projected in the television screen riding at the back of a professional rider. Circuit of the Americas was the speed circuit chosen due to the amount of linked turns and large straights. In this way, riders performed the same movements on a real speed circuit. Participants had to speed up the simulator and then, when taking a turn, decelerate. To performance well, participants had to move their bodies up and down, moving the centre of gravity of the moto-pilot inward and outwards on the simulator to the various degrees required to finish the test. The top inclination of the simulator was 60° and riders had to rest their knees on the floor, like riding on real circuit.

Before starting the test, subjects had the opportunity of accommodation on the motorcycle simulator riding for a period of two-minute. Riders’ accommodation consisted of riding on the simulator, making a first contact on the motorcycle in order to test their adaptation and feeling of comfort with their motorcycle boots on the footpeg. Each rider wore motorcycle boots with a standard template. 

During the test, participants were asked to complete more or less maximum speed simulated in straight-a-ways and at reduced speeds in turns, for a riding period of 20 minutes for every HI test accomplished. Participants rested for five minutes between each 20 minutes testing session for each HI on the simulator. Data were collected from the right leg in right and left turns. The order in which HI were tested was random.

We assessed four types of HI: only polypropylene (58° DShore); polypropylene (58° DShore) with selective aluminium in hallux and metatarsal heads (60HB Brinell hardness); ethylene vinyl acetate (EVA) (52° AShore); and, finally, ordinary EVA (25° AShore) as the control. 

For our research six right leg muscles were tested with EMG: 1) fibularis brevis, 2) fibularis longus, 3) tibialis anterior, 4) gastrocnemius lateralis, 5) gastrocnemius medialis and 5) soleus. Previously, every subject was prepared for testing in a quiet place. Subject skin was depilated and cleaned with 70% alcohol liquid and, later, electrodes were affixed to the six muscle areas. Previously, each subject was prepared for testing in a quiet place. Subjects’ skin was depilated, cleaned with 70% alcohol liquid, and abraded to decrease electrical impedance between electrodes to less than 5 kΩ.

The accurate whereabouts of the seven electrodes (channel E1 to E7) were as follows:

E1: Biceps femoris: middle line since the ischial tuberosity to the lateral epicondyle of the tibia.
E2: Gluteus maximus: middle line between the sacrum to the bigger trochanter.
E3: Gluteus medius: middle line from the iliaca crest to the trochanter.
E4: Semitendinous: middle line since the ischial tuberosity to the medial epicondyle of the tibia.
E5: Tensor fasciae latae: middle line since the anterior-superior spina iliaca to the lateral femoral condyle.
E6: Vastus lateralis: 2/3 on the line since the anterior-superior spina iliaca to the patella lateral side.
E7: Vastus medialis: since the anterior-superior spina iliaca to the joint space of the medial ligament.

**Figure 1:**
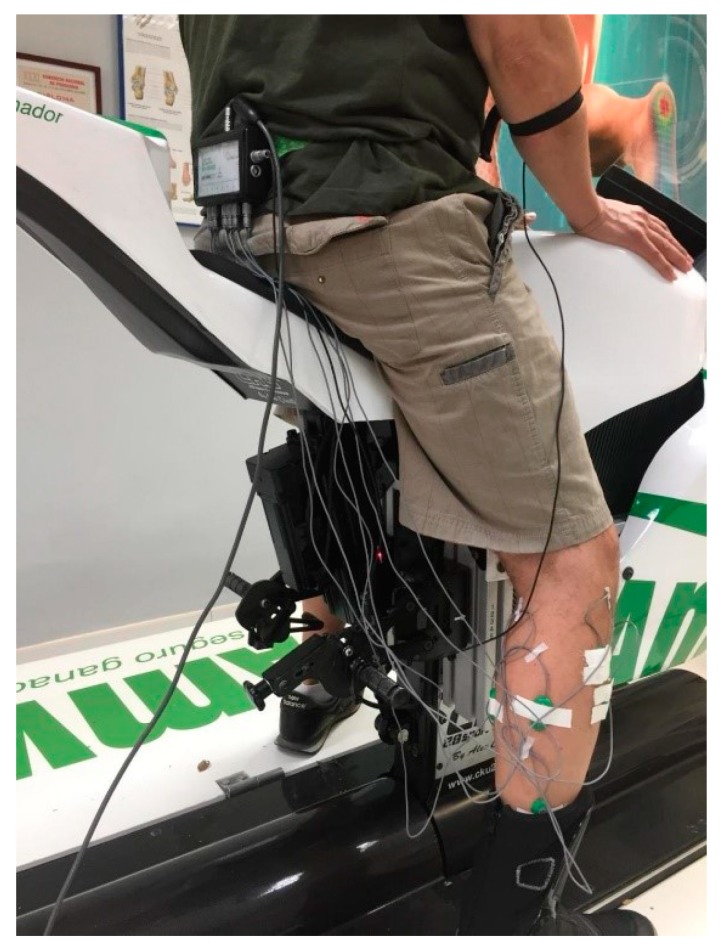
Right EMG electrodes positioning and their channels (E1–E7).

The surface electrodes were placed 2 cm apart (positive and negative) and parallel on the six muscle areas and fixed with hypoallergenic tape to prevent from peeling or moving on the skin (Figure 1) [20].

Each subject performed, for a duration of eight seconds, three maximum voluntary isometric contractions (MVIC), which were recorded for every muscle analysed [21]. This enabled time for the subject to assemble the contraction and for the trial to steady the lower limb. The purpose was to normalize the maximal amplitude. A well-known limitation is the subject’s veracity in performing the MVIC properly [21]. Even knowing that each contraction varies and decreases due to muscle fatigue in an effort, it is better to normalize values in the way described before than not to establish standard values of normality [22]. Subjects rested for a period of time of one or two minutes to decrease the effect of muscular fatigue [23].

The EMG electrodes came out from a light-handed backpack interface, the model was MWX8. The EMG data was recorded with the program DataLINK (Biometrics Ltd., Ladysmith, VA, USA) (version 5.0).

### 2.3. Statistical Analysis

Statistical analyses were performed according to the methods proposed by a prior research study [18]. First, Shapiro–Wilk tests were applied to assess normality of the socio-demographic data and outcome measurements. According to these results, a normal distribution was determined if the *p*-value was ≥ 0.05. 

Second, socio-demographic characteristics, including age, BMI, Spanish foot size, height and weight, were described using standard deviations (SDs) and 95% confidence intervals (95% CIs).

Third, reliability was assessed within trials for each rider by intraclass correlation coefficients (ICCs). According to these analyses, ICCs values were considered as poor (ICCs < 0.40), fair (ICCs = 0.40–0.59), good (ICCs = 0.60–0.74), and excellent (ICCs ≥ 0.75) [24,25]. Reliability coefficients > 0.90 were adequate for clinical measurements as proposed by Portney and Watkins [26]. In addition, means and SDs as well as standard error of measurements (SEMs) were calculated [24]. SEMs were determined according to Bland and Altman’s formula (SEMs = SD × sqrt (1−ICC)) [27].

Fourth, minimal detectable changes (MDCs) were calculated in order to determine the minimal change’s magnitudes to get a 95% CI regarding changes observed between two tests to reflect the true changes and avoid measurement errors [28]. MDCs were calculated according to SEMs values (SEMS = 1.96 × SEM × √2).

Fifth, Cohen’s *d* coefficients were determined in order to report effect sizes for between-groups comparisons. Effect sizes were considered as slight (*d* ≤ 0.20), fair (*d* = 0.20–0.49), moderate (*d* = 0.50–0.79) and large (*d* > 0.79) [29].

Finally, Mann–Whitney *U* tests were used to determine statistically significant differences for between-groups comparisons according to non-parametric data results of the Shapiro–Wilk tests. Regarding all tests, *p*-values < 0.05 were considered as statistically significant. Furthermore, SPSS 19.0 (Windows; IBM; Chicago, IL, USA) was used to carry out all statistical analyses.

## 3. Results

The sociodemographic characteristics of our study are shown in Table 1.

Results of the Shapiro–Wilk test of the HTM using randomly HI types performing left and right turns on the simulator are shown in Table 2. Results for EMG activity in the HTM using random HI types for different turns on the simulator are displayed in Table 3.

Table 4 displays the results of EMG peak amplitude patterns by sort of HI for different turns on the simulator. All muscle reduce peak amplitude patterns with harder insoles except for the tensor fasciae latae in right and left turns. This muscle increases the peak amplitude with herder insoles.

Table 5 display the comparison between couple of HIs on the EMG peak activity patterns. The smallest amplitude peaks results were obtained with the selective aluminium HI compared with the others templates. The value was *p* < 0.001 in all muscle analysed. Nevertheless, polypropylene 58° D Shore HI displayed lesser EMG amplitude peaks than EVA compared with ordinary EVA for all muscles analysed (*p* < 0.001). The results for wearing EVA are lower in comparison with ordinary EVA for all muscles.

## 4. Discussion

The aim of the study was to identify hip and thigh muscles and decrease the activity of muscular patterns using hard insoles while riding on a motorcycle. There are several studies about the influence of lower limb muscles during cycling with EMG [30,31,32,33]. Notably, Bousie et al. analysed the variation of the plantar pressures using templates with different hardness during cycling, concluding that contoured templates raised contact area in cycling [31]. Further, Casado et al. described changes in motoriders’ plantar pressures using different custom foot insoles with different hardness when subjects rode on a motorcycle simulator, resulting in metatarsal foot heads and hallux pressures decreasing with the hardest custom foot insoles [34].

Fortier Guillaume et al. analysed EMG patterns of horse riders and their findings were that biceps femoris, gluteus maximus and rectus femoris had a continuous pattern of activation throughout the cross-country course. They suggest that these muscles are stabilisers and coordinators of the horse rider’s [35].

Speed circuit is composed by straights and multiple turns and bends that requires motoriders a high precise control of the dynamics and the distribution of the centre of mass of the sport motorcycle. The rider achieves stability on the motorcycle with the whole body, with the upper limbs supported by the hands on the handlebars and handles, with the leg resting against the motorcycle fairing and, finally, with the foot resting on the footpeg. Motoriders execute load transfers on the motorcycle in the course of variations in speed and quick tilts on turns that demand repeated physical effort [1,32].

In our research, we found lower HTM activity when the subject’s knee was on the motorcycle, specifically in left cornering, being higher in right cornering, that is to say, when the subject knee was on the ground. This difference may be due to the rider having to lift the motorcycle to the vertical position and the maximum strength does it with the leg that is in contact with the ground. There is a lack of research with respect to measurements regarding the thigh and hip muscles strength of riders. We have found research regarding the influence of upper limb muscles on handgrip strength and lumbar isometric strength [1,2,3,36].

Tomida et al analysed the incidence of injuries in elite competitors but in their research there is a lack of lower limb muscle injury patterns. Most of the injuries were about fractures following ligament injury, but not of muscle injuries [37].

In our study, we analysed the alteration in the EMG patterns in the right hip and right leg of each subject cornering in left and right turns using different HIs. Riders get motorcycle stability taking a turn knocking with the knee on the inside of the turn and, consequently, a great force on the footrest with the medial side of the forefoot is applied. On the other hand, the opposite leg rests on the motorcycle chassis, preparing the stability to put the rider in a neutral position once cornering is finished. Biomechanically, the main role of the tensor fascia latae (TFL) muscle is swinging the weight of the body, and performing hip abduction for the non-weight-holding leg during walking [38]. The TFL performs the most muscle activity in both right and left turns. This is due to its main function in abduction and internal rotations of hip and knee flexor. For these reason the TFL is one of the most significant muscles for maintaining steadiness at the time that the knee touches the floor on the turn. These movements are constantly performed by riders in a turn to the right and left.

The results in our study wearing the select aluminium HI had considerably lesser muscle amplitude peaks than the other HIs for the gluteus maximus; gluteus medius; rectus femoris; biceps femoris; semitendinous; vastus lateralis and vastus medialis. For the TFL the select aluminum HI had considerably lesser muscle amplitude peaks than other HIs in different turns, but considerably higher peak amplitude than other muscles. We suppose that the elevated activity of the TFL in right and left turns, when the knee contacts on road and the knee is close to the motorcycle, increases steadiness in riding. Additionally, reduced activity of the other HTM in turns would probably help to reduced fatigue in riding.

More research in sport motorcycling are necessary to study the biomechanical performance of the hip and lower limb muscles due to the complexity of the movements performed by a rider taking multiple turns and the influence of extrinsic factors, such us gravity force and inertia. Data we obtained in our study could assess the overloading of HTM in such situations. 

A limitation of our study is the lack of inertial forces and steering control challenges in our simulator that occur when riding a motorcycle on a real speed circuit. Further research is needed on the physical effects of motorcycling during actual sport races.

## 5. Conclusions

Using the hardest templates in our study (selective aluminium) the EMG amplitude peaks decreased in all HTM. In order from lower amplitude peaks in HIs were polypropylene 58° D Shore, EVA 52° A Shore, and ordinary EVA 25° A Shore. Likewise, the TFL was the only muscle that maintained a constant contraction in right and left turns, being the main stabilizer in sport motorcycling.

## Figures and Tables

**Table 1:** Sociodemographic characteristics.

	N	Age (years)	Heigh (cm)	Weigh (Kg)	BMI (Kg/m^2^)	Foot Size
Media ± SD	8	33 ± 5.14	175 ± 44	71 ± 44	23.45 ± 1.04	42.22 ± 2.86
(CI 95%)		(29.04–36.96)	(169.77–181.12)	(67.50–75.38)	(22.65–24.26)	(40.02–44.42)

**Table 2:** Normality test of the EMG variables of the thigh and hip muscles for different HI.

Variable HTM and Turn	*p* Value	Variable HTM and Turn	*p* Value
Biceps Femoris ordinary EVA left turn	0.331	Semitendinous polypropylene left turn	0.037
Biceps Femoris ordinary EVA right turn	0.214	Semitendinous polypropylene right turn	0.386
Biceps Femoris EVA left turn	0.836	Semitendinous selective aluminum left turn	0.718
Biceps Femoris EVA right turn	0.724	Semitendinous selective aluminum right turn	0.837
Biceps Femoris polypropylene left turn	0.300	Tensor Fasciae Latae ordinary EVA left turn	0.238
Biceps Femoris polypropylene right turn	0.542	Tensor Fasciae Latae ordinary EVA right turn	0.554
Biceps Femoris selective aluminum left turn	0.053	Tensor Fasciae Latae EVA left turn	0.484
Biceps Femoris selective aluminum right turn	0.616	Tensor Fasciae Latae EVA right turn	0.229
Gluteus Maximus ordinary EVA left turn	0.148	Tensor Fasciae Latae polypropylene left turn	0.651
Gluteus Maximus ordinary EVA right turn	0.340	Tensor Fasciae Latae polypropylene right turn	0.594
Gluteus Maximus EVA left turn	0.918	Tensor Fasciae Latae selective aluminum left turn	0.756
Gluteus Maximus EVA right turn	0.581	Tensor Fasciae Latae selective aluminum right turn	0.009
Gluteus Maximus polypropylene left turn	0.327	Vastus Lateralis ordinary EVA left turn	0.799
Gluteus Maximus polypropylene right turn	0.610	Vastus Lateralis ordinary EVA right turn	0.315
Gluteus Maximus selective aluminium left turn	0.447	Vastus Lateralis EVA left turn	0.559
Gluteus Maximus selective aluminium right turn	0.040	Vastus Lateralis EVA right turn	0.483
Gluteus Medius ordinary EVA left turn	0.547	Vastus Lateralis polypropylene left turn	0.578
Gluteus Medius ordinary EVA right turn	0.049	Vastus Lateralis polypropylene right turn	0.516
Gluteus Medius EVA left turn	0.915	Vastus Lateralis selective aluminum left turn	0.585
Gluteus Medius EVA right turn	0.601	Vastus Lateralis selective aluminum right turn	0.718
Gluteus Medius polypropylene left turn	0.591	Vastus Medialis ordinary EVA left turn	0.005
Gluteus Medius polypropylene right turn	0.399	Vastus Medialis ordinary EVA right turn	0.946
Gluteus Medius selective aluminium left turn	0.143	Vastus Medialis EVA left turn	0.146
Gluteus Medius selective aluminium right turn	0.494	Vastus Medialis EVA right turn	0.531
Semitendinous ordinary EVA left turn	0.272	Vastus Medialis polypropylene left turn	0.763
Semitendinous ordinary EVA right turn	0.341	Vastus Medialis polypropylene right turn	0.928
Semitendinous EVA left turn	0.682	Vastus Medialis selective aluminum left turn	0.025
Semitendinous EVA right turn	0.461	Vastus Medialis selective aluminum right turn	0.054

Abbreviations: EVA: ethyl-vinyl-acetate; Right turn: Knee on the floor; Left turn: Lower extremity towards the motorcycle body part. *p* values are from Shapiro-Wilk test.

**Table 3:** Results of the Intraclass correlation coefficient, standard error of measurement and minimal detectable change for electromyography activity patterns of the Hip and Thigh muscles by types of hardness insoles for left and right turns on the simulator.

HTM and Turn	Ordinary EVA 25°			EVA 52°			Polypropylene 58°			Selective Aluminium 60°		
	25° A Shore			52° A Shore			58° D Shore			(60° Brinell Hardness)		
	ICC	SEM	MDC	ICC	SEM	MDC	ICC	SEM	MDC	ICC	SEM	MDC
	(95%CI)			(95%CI)			(95%CI)			(95%CI)		
**Rectus Femoris left turn**	0.983 (0,948–0.996)	0.275	0.763	0.765 (0.242–0.943)	0.184	0.511	0.849 (0.548–0.963)	0.159	0.442	0.886 (0.635–0.972)	0.155	0.431
**Rectus Femoris right turn**	0.963 (0889–0.991)	0.233	0.645	0.961 (0.876–0.990)	0.164	0.454	0.959 (−0.872–0.990)	0.184	0.511	0.918 (0.746–0.980)	0.226	0.627
**Biceps Femoris left turn**	0.630 (−0.136–0.909)	0.116	0.32	0.843 (0.537–0.961)	0.103	0.286	0.543 (−0.269–0.883)	0.122	0.337	0.093 (−2.018–0.782)	0.143	0.396
**Biceps Femoris right turn**	0.310 (−1.003–0.826)	0.158	0.437	0.331 (−1.198–0.839)	0.147	0.408	0.666 (−0.029–0.918)	0.116	0.32	0.867 (0.603–0.967)	0.124	0.344
**Gluteus Maximus left turn**	0.950 (0.845–0.988)	0.224	0.62	0.781 (0.312–0.946)	0.295	0.817	0.540 (−0.534–0.890)	0.224	0.62	0.564 (−0.268–0.890)	0.231	0.641
**Gluteus Maximus right turn**	0.821 (0.467–0.955)	0.377	1.044	0.548 (−0.583–0.893)	0.289	0.801	0.533 (−0.652–0.890)	0.39	1.080	0.560 (−0.305–0.890)	0.332	0.919
**Gluteus Medius left turn**	0.679 (0.047–0.920)	0.351	0.974	−0.089 (−2.228–0.727)	0.501	1.388	0.695 (0.073–0.925)	0.398	1.102	0.701 (0.076–0.927)	0.366	1.016
**Gluteus Medius right turn**	0.459 (−0.547–0.863)	0.405	1.121	−0.706 (−6.381–0.618)	0.601	1.665	0.821 (0.417–0.957)	0.44	1.220	0.833 (0.458–0.959)	0.356	0.985
**Semitendinous left turn**	0.873 (0.612–0.969)	0.089	0.247	0.620 (−0.213–0.908)	0.136	0.376	0.839 (0.518–0.960)	0.096	0.267	0.614 (−0.202–0.905)	0.118	0.327
**Semitendinous right turn**	0.281 (−1.571–0.831)	0.136	0.376	0.722 (0.192–0.930)	0.116	0.322	0.659 (−0.140–0.917)	0.099	0.275	0.553 (−0.146–0.881)	0.127	0.352
**Tensor Fasciae Latae left turn**	0.898 (0.689–0.975)	0.153	0.425	0.660 (−0.026–0.916)	0.274	0.76	0.965 (0.895–0.991)	0.204	0.565	0.153 (−2.148–0.803)	0.175	0.485
**Tensor Fasciae Latae right turn**	0.564 (−0.369–0.894)	0.158	0.439	0.217 (−0.879–0.786)	0.265	0.736	0.920 (0.756–0.980)	0.164	0.455	0.595 (−0.070–0.894)	0.185	0.512
**Vastus Lateralis left turn**	0.532 (−0.301–0.880)	0.157	0.436	0.473 (−0.687–0.872)	0.145	0.402	0.643 (−0.131–0.913)	0.108	0.298	0.824 (0.440–0.957)	0.143	0.395
**Vastus Lateralis right turn**	0.872 (0.612–0.968)	0.129	0.357	0.861 (0.590–0.965)	0.145	0.403	0.927 (0.772–0.982)	0.105	0.292	0.850 (0.534–0.963)	0.139	0.386
**Vastus Medialis left turn**	0.683 (0.119–0.919)	0.141	0.39	0.301 (−0.367–0.783)	0.142	0.394	0.730 (0.209–0.932)	0.218	0.605	0.858 (0.555–0.965)	0.143	0.397
**Vastus Medialis right turn**	0.972 (0.913–0.993)	0.192	0.533	0.726 (0.149–0.933)	0.241	0.667	0.947 (0.835–0.987)	0.166	0.459	0.720 (0.109–0.932)	0.217	0.601

Abbreviations: ICC, intraclass correlation coefficient; SEM: standard error of the mean; MDC: minimal detectable change; Right turn: Knee on the floor; Left turn: Lower extremity towards the motorcycle body part; EVA: ethy-vinyl-acetate; 95% CI, 95% confidence interval.

**Table 4:** Measurement of the Peak amplitude of the electromyographic patterns of the Thigh and Hip muscles for different HI for left and right turns on the simulator.

	Ordinary EVA 25° 25° A Shore		EVA 52° 52° A Shore		Polypropylene 58° 58° D Shore		Selective Aluminium 60° 60° Brinell Hardness	
HTM and Turn	Mean ± SD	Median	Mean ± SD	Median	Mean ± SD	Median	Mean ± SD	Median
	(95% CI)	(95% CI)	(95% CI)	(95% CI)	(95% CI)	(95% CI)	(95% CI)	(95% CI)
**Rectus Femoris left turn**	14.08 ± 2.11	13.30	6.89 ± 0.38	6.97	5.31 ± 0.41	5.19	5.09 ± 0.46	4.87
	(12.46 to 15.71)	(12.49 to 16.38)	(6.60 to 7.18)	(6.57 to 7.22)	(4.99 to 5.62)	(5.03 to 5.43)	(4.74 to 5.44)	(4.70 to 5.27)
**Rectus Femoris right turn**	86.73 ± 1.21	86.46	86.42 ± 0.83	86.46	79.74 ± 0.91	79.81	73.02 ± 0.79	73.15
	(85.79 to 87.06)	(86.05 to 87.67)	(85.78 to 87.06)	(85.89 to 87.10)	(79.04 to 80.44)	(79.24 to 80.45)	(72.42 to 73.62)	(72.42 to 73.64)
**Biceps Femoris left turn**	20.74 ± 0.19	20.7	20.32 ± 0.26	20.35	19.41 ± 0.18	19.39	18.73 ± 0.15	18.77
	(20.60 to 20.88)	(20.61 to 20.88)	(20.12 to 20.52)	(20.09 to 20.53)	(19.27 to 19.54)	(19.21 to 19.47)	(18.62 to 18.85)	(18.68 to 18.86)
**Biceps Femoris right turn**	24.18 ± 0.19	24.15	23.21 ± 0.18	23.16	21.59 ± 0.20	21.58	20.09 ± 0.34	20.18
	(24.04 to 24.33)	(24.04 to 24.21)	(23.07 to 23.35)	(23.16 to 23.25)	(21.43 to 21.74)	(21.49 to 21.75)	(19.83 to 20.35)	(19.82 to 20.35)
**Gluteus Maximus left turn**	18.12 ± 1.00	18.44	16.73 ± 0.63	16.78	12.14 ± 0.33	12.29	11.11 ± 0.35	11.11
	(17.36 to 18.89)	(17.73 to 18.91)	(16.24 to 17.22)	(16.31 to 17.26)	(11.88 to 12.39)	(11.82 to 12.29)	(10.84 to 11.38)	(10.87 to 11.35)
**Gluteus Maximus right turn**	28.18 ± 0.89	27.9	26.74 ± 0.43	26.71	23.09 ± 0.57	22.93	21.51 ± 0.50	21.75
	(27.50 to 28.87)	(27.42 to 28.84)	(26.41 to 27.07)	(26.71 to 26.95)	(22.63 to 23.50)	(22.70 to 23.40)	(21.13 to 21.90)	(21.04 to 21.99)
**Gluteus Medius left turn**	36.05 ± 0.62	35.87	33.47 ± 0.48	33.33	27.58 ± 0.72	27.3	24.13 ± 0.67	24.13
	(35.58 to 36.52)	(35.56 to 36.19)	(33.11 to 33.84)	(33.33 to 33.65)	(27.03 to 28.14)	(26.98 to 27.94)	(23.61 to 24.64)	(23.81 to 24.13)
**Gluteus Medius right turn**	68.25 ± 0.55	68.25	66.70 ± 0.46	66.67	58.27 ± 1.04	58.1	56.44 ± 0.87	56.51
	(67.83 to 68.68)	(68.25 to 68.57)	(66.35 to 67.06)	(66.35 to 66.98)	(57.47 to 59.07)	(57.46 to 58.73)	(55.77 to 57.10)	(55.87 to 57.14)
**Semitendinous left turn**	26.12 ± 0.25	26.16	25.49 ± 0.22	25.57	24.26 ± 0.24	24.39	23.19 ± 0.19	23.21
	(25.93 to 26.32)	(26.08 to 26.24)	(25.32 to 25.66)	(25.32 to 25.65)	(24.07 to 24.44)	(24.14 to 24.39)	(23.04 to 23.33)	(23.12 to 23.29)
**Semitendinous right turn**	30.16 ± 0.16	30.13	29.69 ± 0.22	29.62	28.80 ± 0.17	28.78	28.42 ± 0.19	28.44
	(30.04 to 30.29)	(30.04 to 30.30)	(29.52 to 29.85)	(29.54 to 29.87)	(28.67 to 28.93)	(28.69 to 28.95)	(28.27 to 28.57)	(28.35 to 28.52)
**Tensor Fasciae Latae left turn**	89.30 ± 0.48	89.4	87.51 ± 0.47	87.67	82.64 ± 1.09	82.67	79.15 ± 0.19	79.2
	(88.93 to 89.66)	(88.99 to 89.70)	(87.14 to 87.87)	(87.36 to 87.77)	(81.80 to 83.48)	(81.96 to 83.59)	(79.00 to 79.30)	(79.00 to 79.31)
**Tensor Fasciae Latae right turn**	88.71 ± 0.24	88,69	87.11 ± 0.30	87,16	80.35 ± 0.58	80,53	79.11 ± 0.29	79
	(88.52 to 88.89)	(88.48 to 88.89)	(86.88 to 87.34)	(86.85 to 87.36)	(79.90 to 80.79)	(79.82 to 80.73)	(78.89 to 79.33)	(78.90 to 79.31)
**Vastus Lateralis left turn**	45.24 ± 0.23	45.17	43.91 ± 0.20	43.88	42.77 ± 0.18	42.74	38.05 ± 0.34	38.12
	(45.06 to 45.42)	(45.09 to 45.34)	(43.76 to 44.07)	(43.80 to 44.12)	(42.63 to 42.90)	(42.74 to 42.82)	(37.79 to 38.31)	(37.88 to 38.28)
**Vastus Lateralis right turn**	92.58 ± 0.36	92.7	91.67 ± 0.39	91.73	88.50 ± 0.39	88.56	83.44 ± 0.36	83.45
	(92.30 to 92.86)	(92.21 to 92.78)	(91.37 to 91.97)	(91.40 to 91.97)	(88.20 to 88.80)	(88.16 to 88.81)	(83.16 to 83.72)	(83.21 to 83.70)
**Vastus Medialis left turn**	12.24 ± 0.25	12,41	11.04 ± 0.17	11,05	9.03 ± 0.42	9,09	7.77 ± 0.38	7,92
	(12.05 to 12.44)	(12.22 to 12.41)	(10.90 to 11.17)	(10.95 to 11.14)	(8.70 to 9.35)	(8.90 to 9.29)	(7.47 to 8.06)	(7.72 to 8.02)
**Vastus Medialis right turn**	95.13 ± 1.15	95.31	93.83 ± 0.46	93.84	86.53 ± 0.72	86.41	82.08 ± 0.41	82,21
	(94.25 to 96.02)	(95.53 to 95.99)	(93.48 to 94.18)	(93.45 to 94.23)	(85.98 to 87.09)	(86.22 to 87.00)	(81.76 to 82.40)	(81.62 to 82.40)

Abbreviations: EVA, ethyl-vinyl-acetate; SD: standard deviation; Right turn: Knee touching the road; Left turn: Lower extremity towards the motorcycle body part; 95% CI: 95% confidence interval.

**Table 5:** Correlation between different kinds of hardness insoles with regard to EMG activity patterns of the Thigh and Hip muscles.

THM and Curve	Standard EVA	EVA	EVA
	Vs	Vs	Vs
	EVA	Polypropylene	Aluminum
	*p* Value	*p* Value	*p* Value
Rectus Femoris left curve	<0.001	<0.001	<0.001
Rectus Femoris right curve	1.000	<0.001	<0.001
Biceps Femoris left curve	0.004	<0.001	<0.001
Biceps Femoris right curve	<0.001	<0.001	<0.001
Gluteus Maximus left curve	0.002	<0.001	<0.001
Gluteus Maximus right curve	0.042	<0.001	<0.001
Gluteus Medius left curve	<0.001	<0.001	<0.001
Gluteus Medius right curve	0.002	<0.001	<0.001
Semitendinous left curve	0.002	<0.001	<0.001
Semitendinous right curve	0.001	<0.001	<0.001
Tensor Fasciae Latae left curve	<0.001	<0.001	<0.001
Tensor Fasciae Latae right curve	<0.001	<0.001	<0.001
Vastus Lateralis left curve	0.001	<0.001	<0.001
Vastus Lateralis right curve	<0.001	<0.001	<0.001
Vastus Medialis left curve	<0.001	<0.001	<0.001
Vastus Medialis right curve	0.067	<0.001	<0.001

Abbreviations: EVA: ethylene vinyl acetate; THM: Thigh hip muscles.

## Abbreviations

EMG	Electromyography
HTM	Hip and thigh muscles
HI	Hardest insoles
EVA	Ethylene Vinyl Acetate
BMI	Body Mass Index
MDC	Minimal detectable change
